# Innovative Syntheses and Reactivity of Propiolamidines

**DOI:** 10.3390/molecules30030708

**Published:** 2025-02-05

**Authors:** Carlos Ginés, Blanca Parra-Cadenas, Fernando Carrillo-Hermosilla, David Elorriaga

**Affiliations:** 1 Departamento de Química Inorgánica, Orgánica y Bioquímica-Centro de Innovación en Química Avanzada (ORFEO–CINQA), Facultad de Ciencias y Tecnologías Químicas, Universidad de Castilla-La Mancha, 13071 Ciudad Real, Spain; carlos.gines@uclm.es (C.G.); blanca.parra@uclm.es (B.P.-C.); 2 Departamento de Química Orgánica, Inorgánica y Bioquímica-INAMOL, Facultad de Ciencias Ambientales y Bioquímica, Universidad de Castilla-La Mancha, 45071 Toledo, Spain

**Keywords:** amidine, propiolamidine, catalysis, heterocycle

## Abstract

Polydentate ligands with nitrogen donor atoms have possibly given rise to the largest group of coordination complexes described. Among these ligands, amidinates represent a nitrogenated version of carboxylates and allow the formation of complexes with most elements in the periodic table, adopting chelate or bridge coordination modes. The precursors of these ligands, amidines, can present an alkynyl group as the substituent of their central atom, R′N=C(C≡CR)NHR′, which provides an additional point of reactivity for these molecules, as well as a different electronic behavior compared to conventional amidines with alkyl groups on the central carbon atom of the amidine group. These propiolamidines have been obtained through classical stoichiometric organic synthesis procedures or, with greater atomic economy, through catalytic procedures based on Main Group, Transition, or Rare Earth metals. This work reviews these synthesis methods, as well as the reactivity in the obtention of new, more complex heterocyclic organic molecules.

## 1. Introduction

**Scheme 1:**
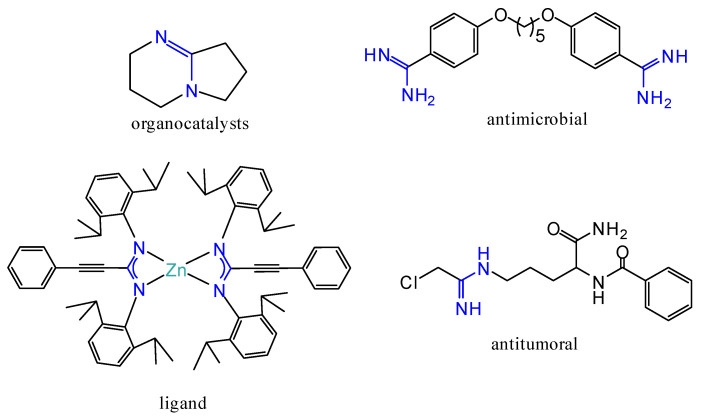
Examples of applications of amidines.

**Scheme 2:**
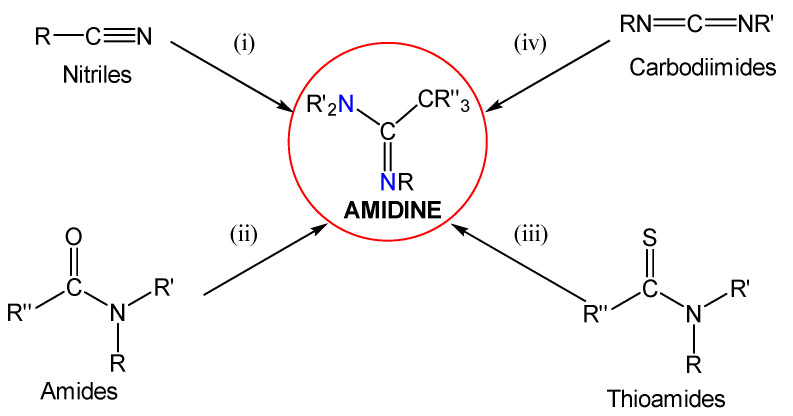
Chemical transformations of different substrates for the synthesis of amidines: (i) e.g., by reaction with amines in the presence of a catalyst; (ii) e.g., by reaction with PCl_5_ and subsequent reaction with amines; (iii) e.g., by condensation with amines; (iv) e.g., by reaction with alkyl or aryl lithium derivatives.

Amidines are well-known organic compounds with the general formula R′N=C(CR^1^R^2^R^3^)NHR′. Since their first synthesis by Gerhardt et al. in 1858 through the reaction of N-phenylbenzimidyl chloride with different anilines [1], they have been extensively studied due to their structural properties and chemical uniqueness. Their potential makes them ideal candidates for applications in synthetic chemistry [2], as biologically and pharmaceutically active species [3,4,5,6,7,8,9,10,11], and as stabilizing ligands for various metal complexes [12,13,14] (Scheme 1). Amidines are typically synthesized from nitriles, which, among other ways, can be transformed into imidic ester salts, which then react with amines to give rise to amidines [15,16,17,18,19,20,21,22,23,24], or catalytically transformed by reaction with amines, with greater atomic economy [25]. Another alternative is to start from amides, which can be chlorinated with PCl_5_ to imidoyl chlorides, which react with secondary or tertiary amines to give rise to amidines [26]. Something similar can be achieved, using thioamides obtained from nitriles by reaction with H_2_S and their subsequent condensation with ammonia or amines [27,28]. Finally, carbodiimides are excellent starting molecules to build amidine molecular architectures by reaction with lithium organometallics [29,30], depending on the substituents present in the backbone of the resulting products (Scheme 2).

**Scheme 3:**
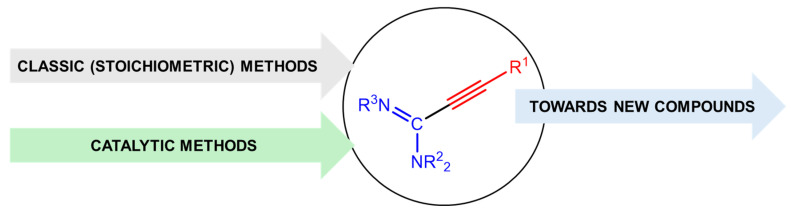
Synthesis and general structure of propiolamidines.

However, analogous compounds where the amidines are conjugated with a C-C triple bond (e.g., R′N=C(C≡CR^1^)NR_2_′) have not been explored extensively. These kinds of amidines are called propiolamidines or propargylamidines. The first report of such compounds dates back to 1971 [31], and, since then, some advances have been made. In this comprehensive review, we present the different synthetic routes known to obtain these compounds and their inherent reactivity to generate new organic products (Scheme 3).

## 2. Synthesis

In the literature, two primary synthetic routes stand out. The first involves stoichiometric synthesis from various starting reagents, while the second offers a more atom-efficient approach through catalytic synthesis. The catalytic route is particularly advantageous as it often requires milder reaction conditions, reduces waste generation, and enhances overall reaction efficiency. This method not only aligns with green chemistry principles but also allows for greater functional group tolerance and scalability, making it a preferred choice for sustainable and industrial applications.

### 2.1. Classical Methods

**Scheme 4:**
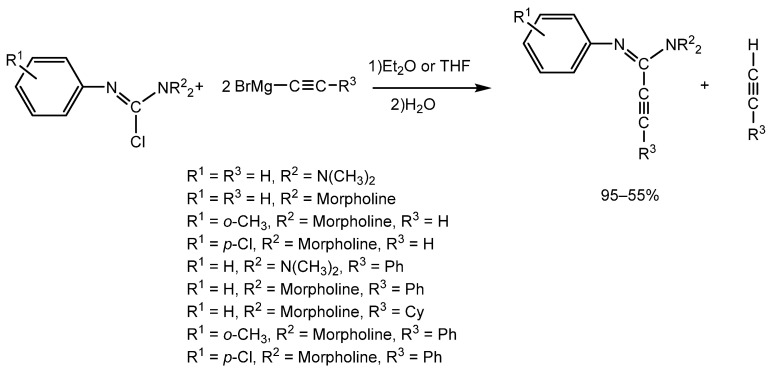
Reaction between chloroformamidines and two equivalents of ethynyl Grignard compounds.

Classical approaches for the synthetic methodologies of these compounds involve the reaction of stoichiometric quantities of terminal alkynyl acetylides as nucleophiles with an electrophile, followed by subsequent hydrolysis to protonate the final compound. Various routes tailored to the nature of the electrophile have been described for synthesizing propiolamidines (or propargylamidines), with most methods emerging in the late 20th century. The first report for the synthesis of these compounds was made in 1971 by Weidemann and coworkers [31]. They found that the treatment of chloroformamidines, as electrophiles, with alkynyl Grignard compounds, as nucleophiles, afforded the amidine derivatives of the corresponding propiolic acid, now known as propiolamidines or propargylamidines, after hydrolysis with water. The reaction of N-aryl-substituted chloroformamidines with two equivalents of ethynyl magnesium bromide in aprotic solvents (Et_2_O, THF) gave the corresponding propiolamidines in moderate to good yields after hydrolysis with water (Scheme 4).

**Scheme 5:**
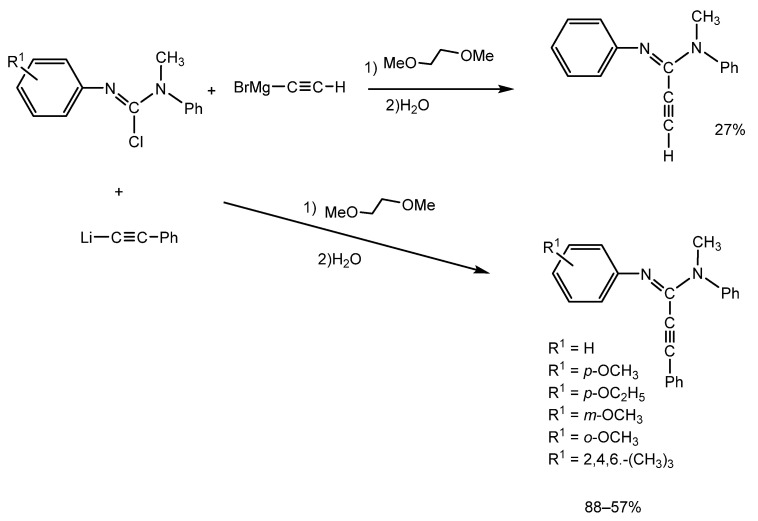
Reaction of N-methyl-N,N′-diphenylchloroformamidines with ethynyl Grignard or lithium phenylacetylide.

Later, this idea was extended to a less basic N-methyl-N,N′-diphenylchloroformamidines [32]. The reaction with ethynyl Grignard in a 1:1 ratio gave propargylamidine in very low yield (~27%). Thus, the Grignard was replaced by lithium phenylacetylide with greater nucleophilic character. In this manner, the reaction of the chloroformamidines with the organolithium reagent in 1,2-dimethoxiethane and, after hydrolysis with water, produced the corresponding amidines in better yields (Scheme 5). Furthermore, in 1979, the same group extended this study to the chloroformamidine where the amine group was swapped by a morpholine motif and the amidic group was substituted by methyl(phenyl)sulfane, 3,4,5-tri(methoxyphenyl), or 4-ethoxynaphthalenyl groups [33]. Giving a twist to this synthetical route, Himbert et al. described the reaction of more nucleophilic ((methyl)(phenyl)amino)ethynyl lithium with a chloroformamidine substituted with N,N-dimethylamino and tosylamide groups [34].

**Scheme 6:**
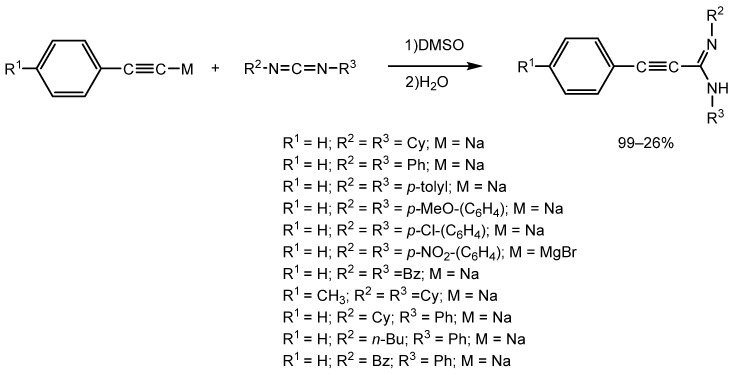
Reaction between metal phenylacetylides and carbodiimides.

Another interesting route for synthesizing propiolamidines mirrors the method described above but substitutes the nucleophile chloroformamidine with a carbodiimide. In this vein, Fujita and coworkers proposed the formation of sodium phenylacetylides by the reaction of metallic sodium and the corresponding acetylides in xylene, in the presence of tertiary amines to stabilize the sodium acetylide. Th subsequent addition of a carbodiimide in the presence of DMSO produces the nucleophilic attack of the sodium salt on the electrophilic carbon atom of the heterocumulene. Finally, after hydrolysis with water, the corresponding propiolamidine is obtained. However, in the case of N,N′-bis(*p*-nitrophenyl)phenylpropiolamidine, this reaction was not successful, requiring the use of the Grignard reagent instead of the corresponding sodium acetylide (Scheme 6) [35].

**Scheme 7:**
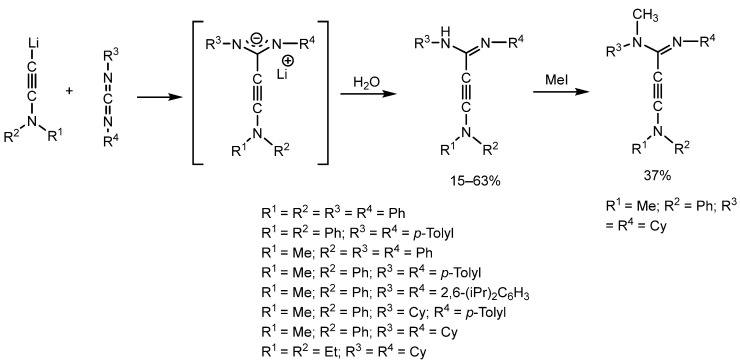
Reaction between lithium aminoacetylides and carbodiimides.

Later, in 1984, Himbert et al. [36] extended this methodology to the use of aminoacetylene reagents instead of phenylacetylenes. Thus, the reaction of one equivalent of lithium aminoacetylides with an equivalent of carbodiimides gave the corresponding ynamines (3-aminopropiolamidines) through a lithium aminopropiolamidinate intermediate. Additionally, this intermediate can be directly transformed into a highly substituted aminopropiolamidine by adding MeI (Scheme 7).

**Scheme 8:**
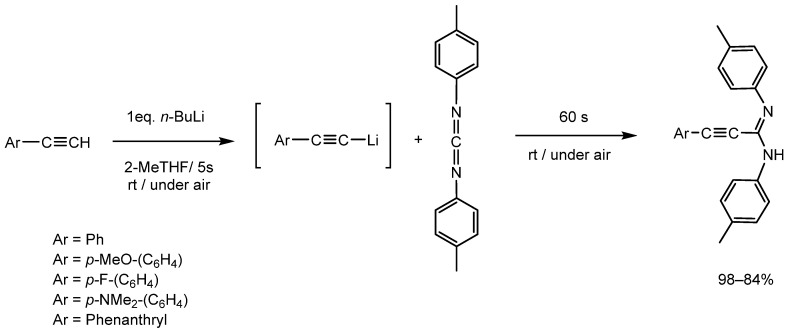
Green approach to obtain propargylamidines by reaction of lithium acetylide generated in situ and di-para-tolylcarbodiimide.

Usually, the employment of these highly polar organometallic compounds requires dry aprotic solvents, low temperatures, and a rigorous inert atmosphere to avoid decomposition reactions and side reactions and for safety (as they could catch fire under normal conditions). However, following this procedure of reacting a lithium acetylide with carbodiimides for the formation of propalgylamidines, but considering the demands of our new era, a green approach was reported by our group. This environmentally friendly methodology is based on the addition of equimolar quantities of n-butyl lithium to phenylacetylenes for 5 s in untreated 2-MeTHF (a sustainable solvent) and the subsequent addition of di-para-tolylcarbodiimide. After stirring the reaction for 60 s at room temperature under normal atmospheric conditions, it was quenched with a saturated aqueous ammonium chloride solution to generate the corresponding phenylpropargylamidines (Scheme 8). The whole process was carried out under bench-type conditions [37].

**Scheme 9:**
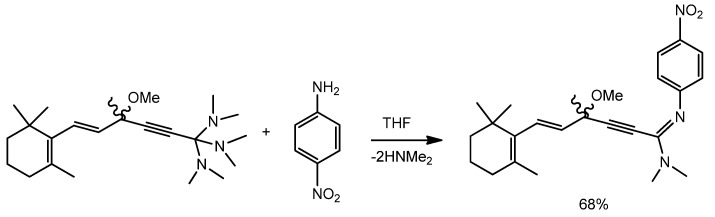
Reaction of the orthoamide with nitroaniline.

Finally, it is known that orthoamides of alkynecarboxylic acids can react with N-H acidic compounds. An especial example relies in this fact: Kantlehner et al. [38] found that the treatment of 3-methoxy-3-methyl-6,6,6-tris(dimethylamino)-1-(2,6,6-trimethyl-1-cyclohexenyl)hex-1-en-4-in with an equimolar quantity of *para*-nitroaniline in dry THF produced the corresponding propioamidine with a concomitant release of dimethylamine (Scheme 9).

### 2.2. Catalytic Methods

In contrast to the stoichiometric methods described above, the catalytic synthesis of propiolamidines is based on a simple addition reaction. Specifically, this process is known as a catalytic hydroacetylenation or hydroalkynylation of carbodiimides, where a terminal alkyne is added to the central carbon of a carbodiimide. This is the simplest method for producing propiolamidines and reported examples of this procedure are numerous. Therefore, the following sections are divided according to the block of the periodic table to which the metal center of the catalyst belongs.

#### 2.2.1. Main Group Element Catalysts

**Scheme 10:**
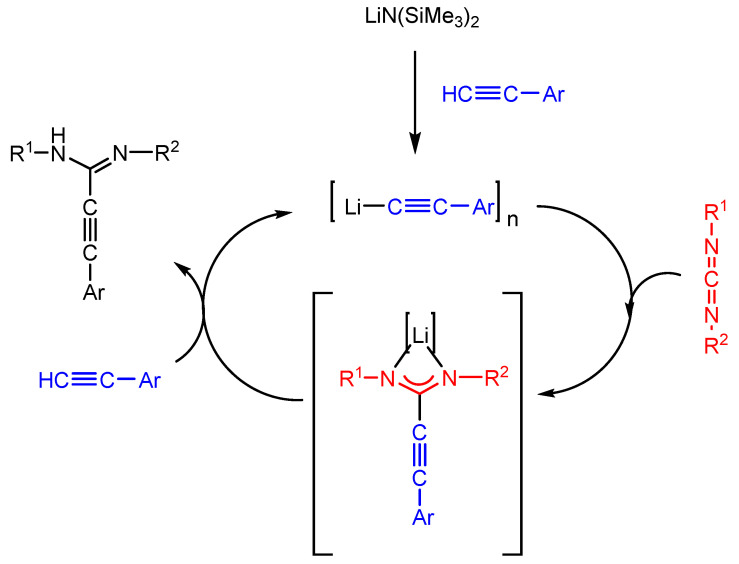
Proposed mechanism for the hydroalkynylation catalyzed by LiN(SiMe_3_)_2_ (**1**).

The first report of the catalytic synthesis of propiolamidines by a main group catalyst was published in 2006 by Richerson and co-workers [39]. It is based on the readily available lithium hexamethyldisilazide (LiN(SiMe_3_)_2_) (**1**) as catalyst precursor. They found that the reaction of several substituted terminal acetylenes with carbodiimides with a 5 mol % catalyst loading for less than two hours at 80 °C produced the desirable amidine. However, the primary limitation of this catalyst was its reaction with diphenylcarbodiimide, as it only functions with aliphatic ones. Moreover, they suggested that other organolithium compounds (*n*-BuLi) and lithium amides worked in the same manner as LiN(SiMe_3_)_2_. Regarding the mechanism, it was proposed that initially the reaction between the lithium amide and acetylide takes place to form the lithium acetylide, followed by carbodiimide insertion to give the lithium amidinate. Transfer protonation from another molecule of acetylene releases the concomitant propioamidine, regenerating the catalytic active species (Scheme 10).

Getting deeper into the understanding of this mechanism [40], they studied by DFT calculations the two critical steps in this cycle: the carbodiimide insertion to form the lithium amidinate and the proton transfer to release the amidine and regenerate the active species. As a model of study, they focused on a TMEDA lithium adduct. Whereas the carbodiimide insertion step was found to happen readily, the critical step was the proton transfer from an acetylene molecule to the released amidine. This proton transfer step takes place through a transition state where one of the nitrogen atoms of the amidinate dis-coordinates to the metal center to accept the proton from the acetylene molecule and form the new lithium acetylide. This process approximates the *sp* carbon atom to the lithium center. However, the bond formed between the alkyne and the lithium is weak and has more contribution from the *sp*-hybridized orbital of the C-H bond rather than the acetylene π-orbital, making it more energetically demanding than the carbodiimide insertion step. Intending to study further, authors tried to extend this study to aluminum amides, but they found that the π-system of the alkynes was not able to form a bond with this metal.

**Scheme 11:**
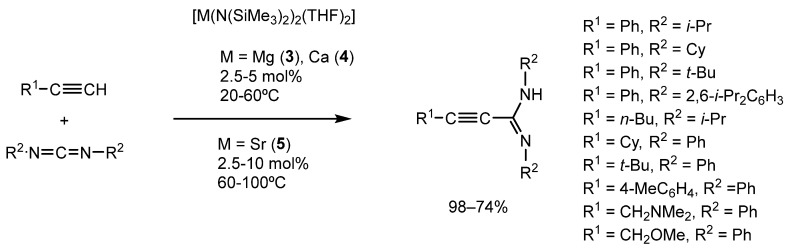
Synthesis of propiolamidines catalyzed by homoleptic complexes of magnesium, calcium, and strontium.

Following this mechanism, several contributions within group 2 have been made. The first one was the application of a β-diketiminate calcium amide complex [{(2,6-(*i*-Pr)_2_C_6_H_3_)NC(Me)CHC(Me)N(2,6-(*i*-Pr)_2_C_6_H_3_)}Ca{N(SiMe_3_)_2_}(THF)] (**2**) in the catalytic reaction of phenylacetylene and diisopropilcabodiimide in ratio 1:1, in toluene at 80 °C for 14 h with 5 mol % of catalyst loading [41]. Thereafter, the study was extended to homoleptic hexamethyldisilazides complexes of magnesium (**3**), calcium (**4**), and strontium (**5**), and to the barium “ate” complex [BaK{N(SiMe_3_)_2_}_3_] (**6**) (Scheme 11) [42]. A trend was observed where the activity increased with the cation size, with the barium complex being the most active even at room temperature. Another peculiarity of these assays was the influence of the solvent system. Whereas the magnesium-catalyzed reaction was significantly inhibited by the addition of THF (9:1 C_6_D_6_:d_8_-THF), the cases with calcium and strontium increased the reactivity. Moreover, this procedure tolerates well aromatic and aliphatic substituents both in carbodiimides and acetylenes.

**Scheme 12:**
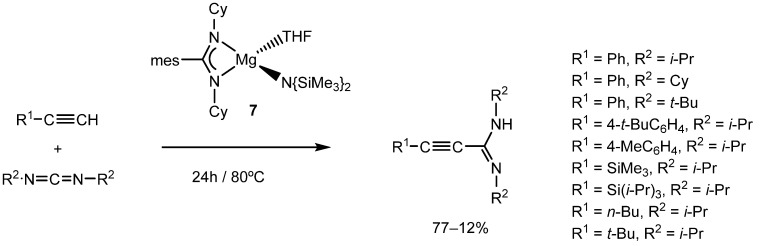
Synthesis of propiolamidines catalyzed by a magnesium amidinate.

On the other hand, Coles reported that an amidinate amide magnesium complex **7** catalyzed the reaction of carbodiimides with terminal alkynes at 80 °C for 24 h and with 1 mol % catalyst loading [43]. However, this procedure presented the limitation of lowering the yield as the steric profile in the carbodiimide increased and completely shut down the reaction with aromatic ones (Scheme 12). Additionally, this study was extended to other commercially available Mg(II) compounds, including Grignard reagents. Whereas both MgBu_2_ and Mg(N{SiMe_3_}_2_)_2_ were active with similar performance as that obtained with the amidinate magnesium complex, the Grignard reagents MgMeBr and MgPhBr gave lower conversion, and MgBr_2_·Et_2_O was inactive in catalysis, suggesting that the activity decreases with the presence of Mg-Br bonds. Furthermore, all evidence proposed that this procedure follows the mechanism of carbodiimide insertion into the M-acetylide bond (performed by the reaction of the precatalyst and acetylene) and subsequent proton transfer mechanism.

**Scheme 13:**
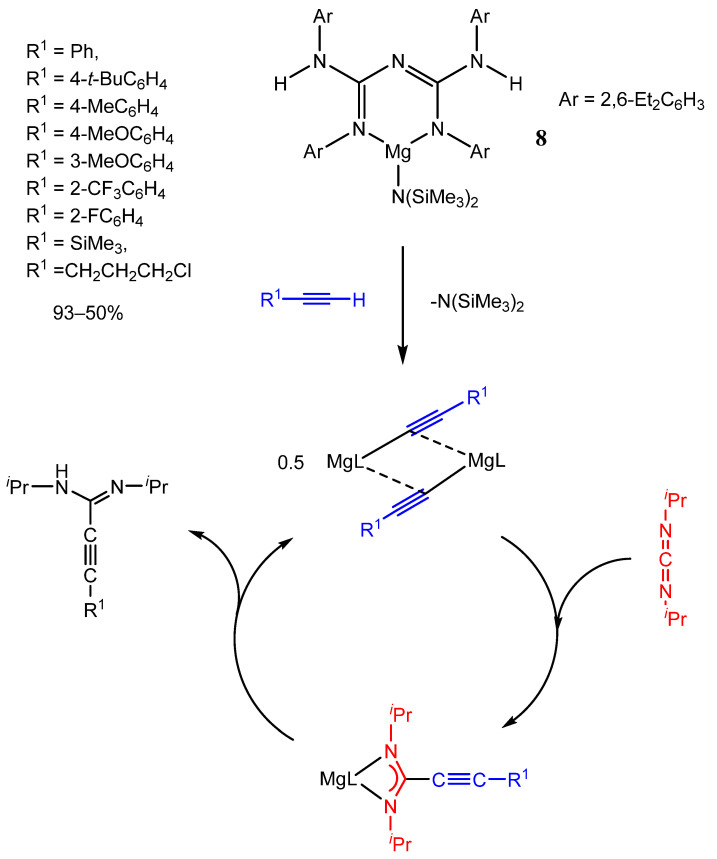
Mechanism for the reaction catalyzed by **8**.

More recently, Nembenna and colleagues found that a bis-guanidinate-supported magnesium (II) amido complex (**8**) promoted the same reaction in 18 h at 90 °C with 3 mol% loading [44]. This precatalyst works smoothly for a range of aliphatic and aromatic acetylenes *o*-, *m*-, and *p*-substituted with electron withdrawing and electron donating groups. Regarding the carbodiimide, only diisopropylcarbodiimide was tested. Through several experiments, authors suggest that the mechanism followed begins with the formation of an acetylide dimer and the subsequent insertion of the carbodiimides giving a porpilamidinate intermediate, which reacts again with alkyne to regenerate the active species (Scheme 13).

**Scheme 14:**
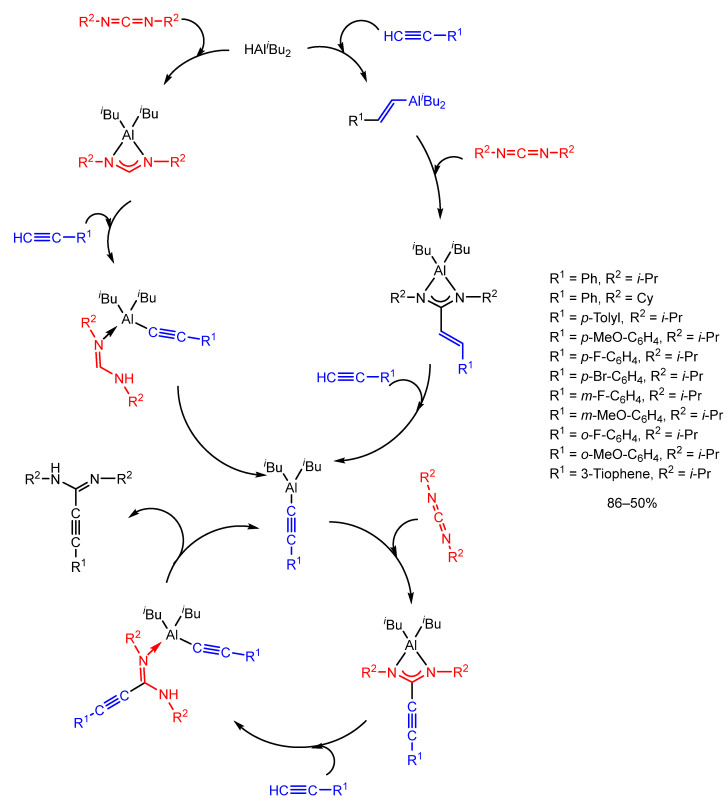
Mechanism for the reaction catalyzed by HAl(*i*-Bu)_2_.

Moving onto group 13, only one example is known for the catalytic synthesis of propaiolamidines [45]. Yang and collaborators tried various organoaluminum compounds as precatalysts in the reaction of phenylacetylide and diisopropylcabodiimide, with HAl(*i*-Bu)_2_ (**9**) being the most active at 80 °C for 36 h in toluene. When the scope was extended, it was found that this procedure failed in the use of aromatic carbodiimides. Conversely, as previously mentioned, earlier DFT studies by Richerson demonstrated that aluminum could not form the acetylide intermediate. This was confirmed in this study during the mechanistic elucidation, where the stoichiometric reaction between the aluminum precursor and phenylacetylene produced (E)-diisobutyl(stytyl)aluminum very slowly at room temperature. However, when the carbodiimide is added to the precatalyst, the insertion into the Al-H bond took place. Subsequent studies suggested that the mechanism is illustrated in Scheme 14.

Firstly, the precatalyst reacts with one molecule of carbodiimide to generate the amidinate species. This species can then react with a molecule of acetylene to form the acetylide complex. In the next step, the acetylide complex inserts one molecule of carbodiimide, resulting in the formation of a propioamidinate complex. This complex subsequently liberates propiolamidine by reacting with another molecule of acetylene, forming the active acetylide species.

Simultaneously, the precatalyst **9** can react with one molecule of acetylene to produce the (E)-diisobutyl(stytyl)aluminum compound. This compound can undergo the insertion of a molecule of carbodiimide to produce an amidinate intermediate, which, by reacting with a molecule of acetylene, generates the active acetylide species.

**Scheme 15:**
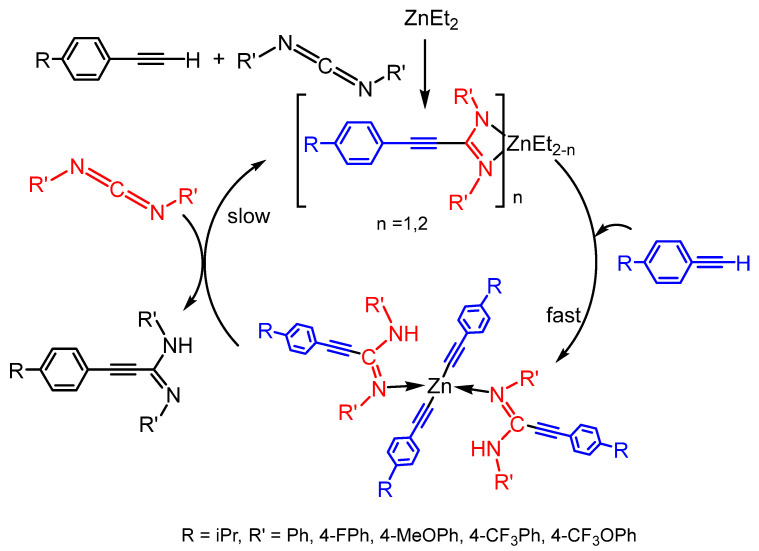
Mechanism for the reaction catalyzed by ZnEt_2_.

Later, our research group pioneered the use of a simple and versatile zinc compound as catalyst in carbodiimide hydroalkynylation [46]. What we found was that 3 mol% of ZnEt_2_ (**10**) was able to catalyze the equimolar reaction of terminal alkynes with carbodiimides in toluene-d_8_ at 70 °C for 24 h. This catalyst tolerates different aliphatic carbodiimides as well as aromatic acetylenes *para*-substituted with electron-withdrawing or -donor groups. During the consecution of the mechanistic studies, it came to light that the plausibly active species was a zinc bispropiolamidinate. The fast step is the addition of two molecules of alkyne and the concomitant discoordination of one nitrogen atom of each amidinate, and the slow one is the insertion of two carbodiimide into the alkynyl-Zn bonds to regenerate the bisamidinate and liberation of two molecules of propiolamidine (Scheme 15).

More recently, another zinc compound has been postulated as precatalyst for this transformation [47]. The dinuclear bisguanidinate-supported zinc hydride, [{LZnH}_2_; L = {(ArHN)(ArN)-C=N-C=(NAr)(NHAr); Ar = 2,6-Et_2_-C_6_H_3_}] (**11**), catalyzes the addition of acetylenes to carbodiimides with a 3 mol% loading at 70 °C for 24 h in deuterated benzene. This procedure works with either aliphatic or aromatic acetylenes and diisopropilcarbodiimide and ditertbutylcarbodiimide. In this case, the proposed mechanism was the standard one: the formation of an acetylide active species and the subsequent insertion of carbodiimide to finally release the propiolamidine by a slow proton transfer from another acetylene molecule, regenerating the active species.

#### 2.2.2. Transition Metal Catalysts

This block is mainly dominated by late transition metals. The first report of using a catalyst based on transition metals is dated from 1988 and it is a binary system of H_4_Ru_4_(CO)_12_ (**12**) and Co_2_(CO)_8_ (**13**) [48]. Only two examples of propiolamidines were synthesized, requiring the reaction of equimolar quantities of diisopropylcarbodiimide and either phenyl- or tert-butylacetylene at 120 °C for 24 h in THF with a 1 mol% catalyst loading. 

**Scheme 16:**
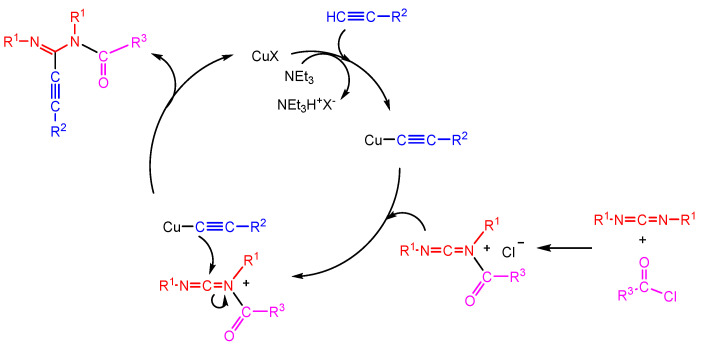
Proposed mechanism for the multicomponent reaction catalyzed by CuI.

On the other hand, in 2008, a copper catalyst was reported for the catalytic synthesis of functionalized propiolamidine derivates [49]. However, in this case, the reaction was pushed a step forward, introducing a multicomponent effect. In that sense, terminal alkynes, acid chlorides, and carbodiimides were efficiently transformed into the corresponding amidines. The reaction of terminal alkynes with carbodiimides and acid chlorides in the ratio 1:1.2:1.2 was catalyzed by the simple copper salt CuI in the presence of two equivalents of triethylamine as an external base. The best reaction conditions were found to be 10 mol% of CuI in acetonitrile for 2–4 h. In this case, the mechanism is slightly different. The first step of acetylide formation must also occur, but, in this case, it is assisted by TEA, which abstracts iodine. Once the acetylide is formed, the insertion reaction occurs, but, instead of a carbodiimide, this time it involves a preformed amide, which results from the reaction between the carbodiimide and the acid chloride (Scheme 16). This procedure is effective for aliphatic carbodiimides, aliphatic alkyniles, and aromatic alkyniles substituted in *para* position with electron-donor and -withdrawing groups and aliphatic and aromatic acid chlorides.

**Scheme 17:**
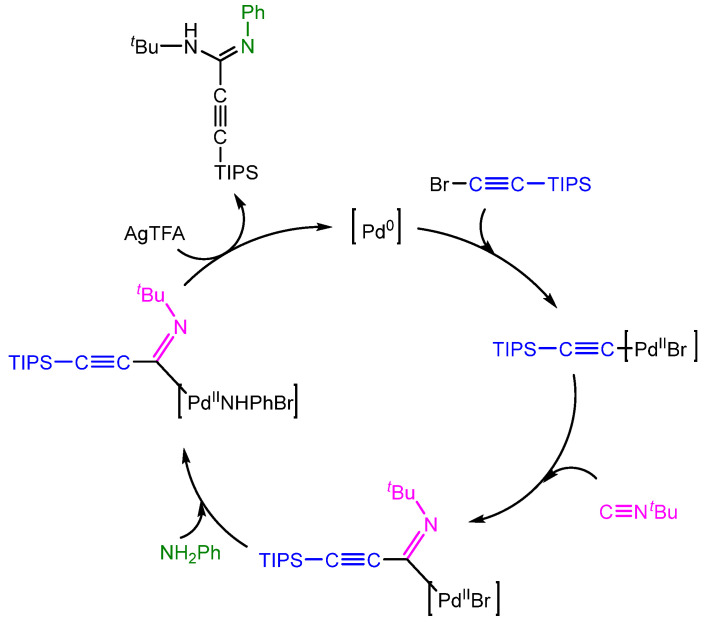
Proposed mechanism for the multicomponent catalyzed by palladium.

Following this multicomponent reaction for the formation of substituted propiolamidines, recently, a new palladium-catalyzed protocol was reported [50]. In this case the components of the reaction are haloalkynes, amine, and isocyanide. This reaction required an amine, the haloalkyne of triisopropyilsilane (TIPS), and isocyanide in a ratio 1:1.5:1.25, PdCl_2_ as precatalyst in 10 mol%, AgTFA in 20mol% as additive, and two equivalents of K_2_CO_3_ as a base in acetonitrile at room temperature for 4 h. On the basis of some experiments and previous studies, authors proposed the catalytic cycle shown in Scheme 17. Firstly, an oxidative addition of bromoalkyne to Pd(0) species gives the alkynylpalladium intermediate. The subsequent migratory insertion of isocyanide affords the imidoyl Pd(II) species, which reacts with aniline, producing the imidoyl amido Pd(II) compound. Finally, by a reductive elimination assisted by AgTFA, the propiolamidine is liberated and the Pd(0) species are regenerated.

**Scheme 18:**
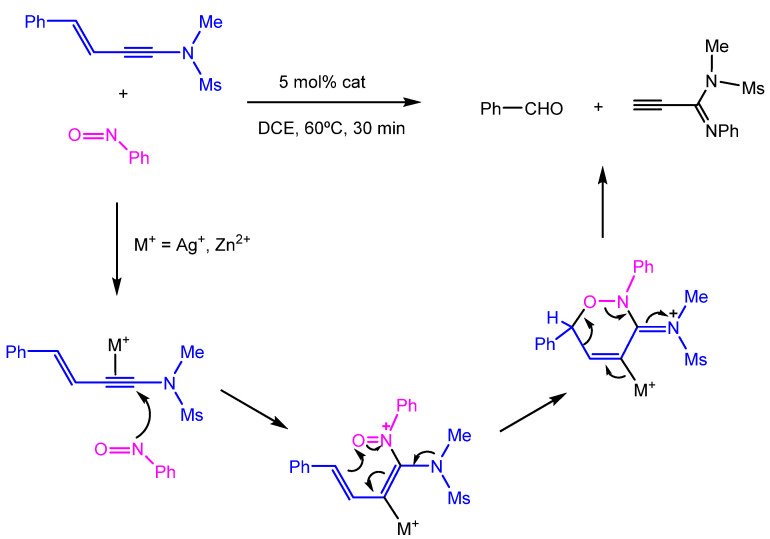
Proposed mechanism for the 1,4-metathesis reaction catalyzed by Zn and Ag.

Another interesting route to synthesize these innovative propiolamidines is by 1,4 metathesis reaction between 3-en-1-ynamides and nitrosoarenes, using different compounds of Zn(OTf)_2_ or AgOAc as precatalysts [51]. Unlike other catalytic procedures, in this case, a mixture of products is obtained, with the majority product corresponding to propiolamidine (Scheme 18). Moreover, the 2-propynimidamides can be converted into the alkynol derivates in a tandem one-pot procedure. The optimal conditions for this reaction are a catalyst loading of 5 mol% and a 1:1.5 ratio ynadine:nitrosoarene, in dichloroethane (DCE), at 60 °C for 30 min.

#### 2.2.3. Rare Earth Metal Catalysts

**Scheme 19:**
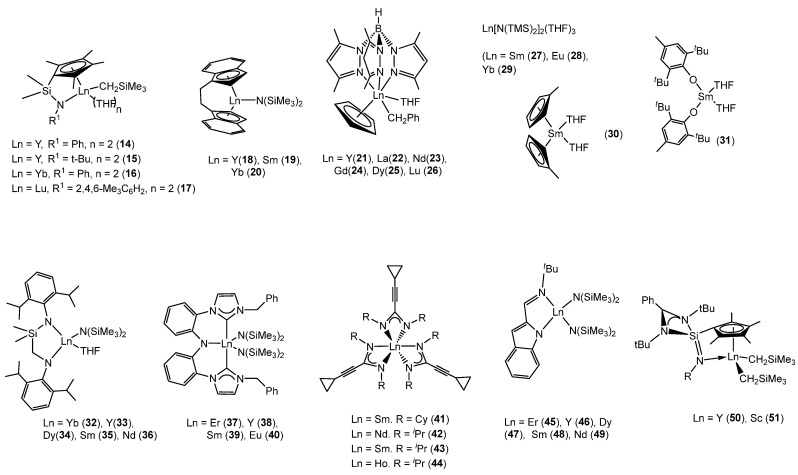
Rare earth catalysts used in the synthesis of propiolamidines.

**Scheme 20:**
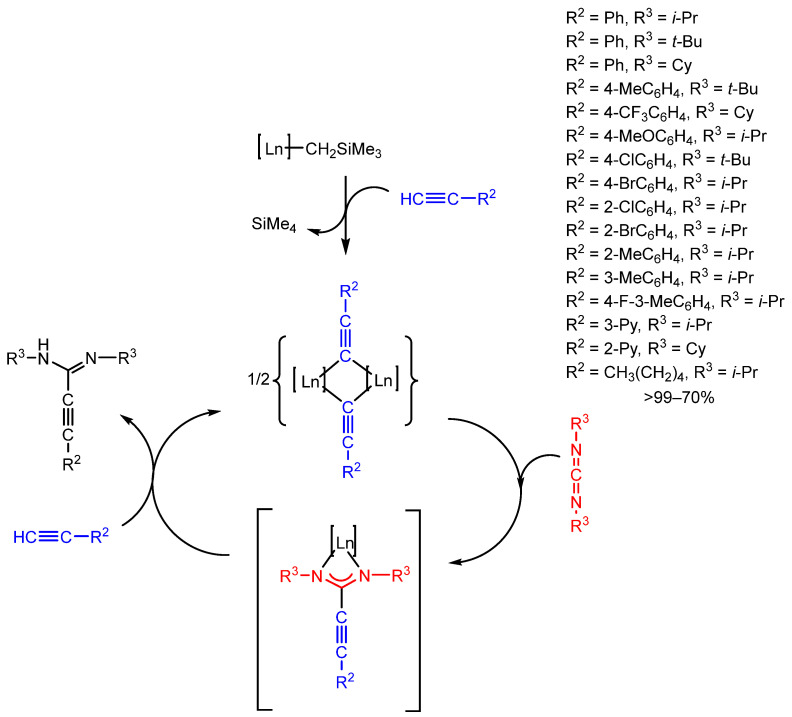
Mechanism proposed for half-sandwich rare earth metal catalysts **14**–**17**.

Despite their relatively low availability, compounds of these metals have been the most employed as catalysts for the synthesis of propargylamidines by far. The first example was reported by Hou and co-workers [52,53], where several half-sandwich rare earth metal complexes bearing silylene-linked cyclopentadienyl-amido ligands acted as excellent catalysts, with yttrium being one of the best among all of them (Scheme 19). The addition of terminal alkynes to carbodiimides takes places with a 3 mol% loading of any of the catalysts in THF, at 80 °C for aromatic alkynes and 110 °C for aliphatic ones. This procedure is successful for aliphatic carbodiimides and tolerates well either electron-withdrawing or -donating groups in the aromatic group of the alkyne, as well as heterocycle alkynes (Scheme 20). The proposed mechanism for these catalysts starts with the formation of an acetylide active species (a dimer in this example), which inserts one molecule of carbodiimide to form a propiolamidinate intermediate. Then, by proton transfer from another molecule of acetylene, the active species is regenerated and the amidine is released.

Keeping the focus on the cyclopentadiene lanthanide compounds, a series of lanthanocene amido complexes were reported to be good catalysts for the hydroalkynylation of carbodiimides with aromatic acetylenes. Complexes (EBI)LnN(TMS)_2_ (EBI = ethylenebis(endine); Ln = Y (**18**), Sm (**19**), Yb (**20**)) (Scheme 19) perform the reaction at 80 °C in THF and with a 2 mol% loading. This system tolerates electron-withdrawing and electron-donating groups in the *para*-positions of the aryl groups. Regarding the carbodiimides, only aliphatic ones were tested. Regarding the mechanism, it was proposed to be identical to the one shown above, with the active species being monomeric.

Additional studies about the use of lanthanide catalysts bearing Cp rings were made by Zhou. Complexes of Y [54,55] (**21**), La [56] (**22**), Nd [57] (**23**), Gd [58] (**24**), Dy [59] (**25**), and Lu [60] (**26**) with mixed Tp^Me2^/Cp ligands (Scheme 19) resulted as good catalysts for this transformation, although Gd catalyst **24** showed the best activity of them. This catalyst is active with aliphatic and aromatic and symmetrical and unsymmetrical carbodiimides, as well as aromatic, heteroaromatic, and aliphatic terminal mono- and dialkynes with electron-withdrawing and electron-donating groups. Again, the mechanism suggested is the standard one proposed for this transformation.

Following the idea that divalent lanthanides complexes are efficient single-electron transfer reagents, the Shen research group tested a bunch of divalent rare earth metal complexes in the reaction for the addition of terminal alkynes to carbodiimides [56]. The simple complexes Ln[N(TMS)_2_]_2_(THF)_3_ (Ln = Sm (**27**), Eu (**28**), Yb (**29**)), (MeC_5_H_4_)_2_Sm(THF)_2_ (**30**), (ArO)_2_Sm(THF)_2_ (ArO = [2,6-(*^t^*Bu)_2_-4-MeC_6_H_2_]) (**31**) (Scheme 19) showed good activity at 1 mol% loading in THF at 80 °C for 3 h. In this case, electron-withdrawing and -donor groups in the phenyl- and naphthyl-substituted rings of the terminal alkyne are well-tolerated in the addition reaction to symmetrical aliphatic carbodiimides. No differences were proposed in the postulated mechanism for these complexes.

As part of the interest in studying rare earth metal amides as catalysts for the synthesis of propiolamidinates, in 2010, Wang et al. [57] reported a family of tetracoordinated lanthanide complexes with the general formula [{(CH_2_SiMe_2_){(2,6-*^i^*Pr_2_C_6_H_3_)N}_2_}Ln{N(SiMe_3_)_2_}(THF)] (Ln = Yb (**32**), Y (**33**), Dy (**34**), Sm (**35**), Nd (**36**)) (Scheme 19). These catalysts were able to produce the amidines by the reaction of equimolar quantities of terminal alkyne and carbodiimide, at 3 mol% in THF at 60 °C for 12 h. Although all of them catalyzed the reaction with good performance, the best one was the samarium complex **35**. Under these conditions, **35** was able to modify aliphatic carbodiimides with aromatic acetylenes substituted with electron-withdrawing and -donor groups. However, with aliphatic acetylenes, it could only achieve moderate yields. The acetylide formation/carbodiimide insertion/proton transfer mechanism was proposed for this catalyst as well.

Later, the same authors reported the first catalysts able to generate the propiolamidinates by the insertion of terminal alkynes into carbodiimides at room temperature. These catalysts are CNC-pincer rare earth metal complexes, bearing diarylamido-linked bis(NHC) ligands **37**–**40** (Scheme 19) [58]. The addition reaction was carried out at room temperature, with equimolar amounts of the reagents and 5 mol% of catalyst loading in THF for 12 h. All the catalysts (**37**–**40**) seemed to have the same activity, indicating that, in this case, the metal ion has no influence on the reaction. These catalysts tolerated well aliphatic carbodiimides and alkynes as well as electron-withdrawing and -donor groups in the *para*-position of the aromatic groups in acetylenes.

As described above, propiolamidinate complexes are sensible intermediates in the catalytic cycle of the addition of terminal alkynes to carbodiimides. Thus, in 2015, several homoleptic propiolamidinate lanthanide complexes (**41**–**44**) were explored as precursors for this reaction (Scheme 19) [59]. Although all complexes catalyzed the reaction, the best performance was obtained with samarium tris(cyclopropylethinyl)amidinate **43**. The reaction was performed at 60 °C in THF with 1 mol% catalyst for 30 min. Aliphatic carbodiimides were tolerated well, as well as aromatic and aliphatic acetylenes.

**Scheme 21:**
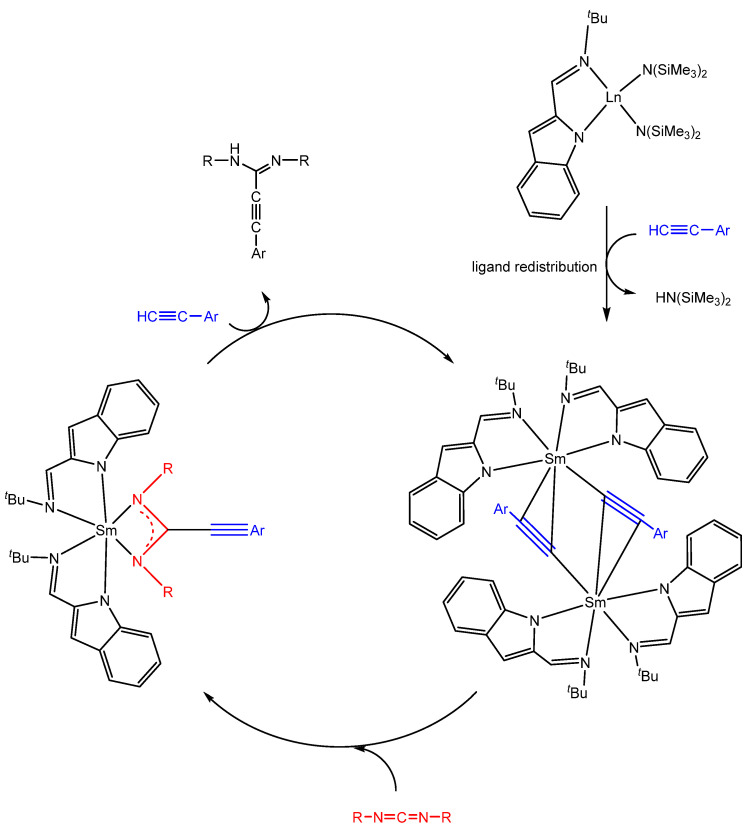
Proposed mechanism for **48**.

More recently, a series of functionalized indolyl lanthanide amido complexes (**45**–**49**) were reported as catalysts for this transformation (Scheme 19) [60]. Although all the complexes catalyzed the addition reaction of terminal alkynes to carbodiimides, samarium compound 46 was the most effective catalyst for this transformation. The optimal conditions were 3 mol% catalyst, THF as solvent, at 60 °C for 12 h. This catalyst was effective with aliphatic and bulky aromatic carbodiimides, as well as aromatic acetylenes para-substituted with electron-withdrawing and -donor groups. Regarding the mechanism, and based on some experimental results, the authors suggested that the first step involves an acid–base reaction of the amide complex with the acetylene, producing a dimeric complex with ligand redistribution. This results in each metal center having two ligands and one alkynyl ligand in an η¹ fashion, with two carbon atoms of the triple bond coordinated to another metal (Scheme 21).

Finally, highly active yttrium and scandium dialkyl complexes (**50**–**51**) can be stabilized by silaimine-functionalized cyclopentadienyl ligands, showing potential for C(sp^3^)−H bond activation, C=N double-bond insertion, and excellent functional group tolerance in catalytic reactions, highlighting their promise in rare earth chemistry [61].

## 3. Reactivity

As described in the synthesis section, the reactivity of propiolamidines can be categorized into two distinct areas. The first area encompasses classical approaches, while the second area is centered on catalytic transformations.

### 3.1. Classical Methods

**Scheme 22:**
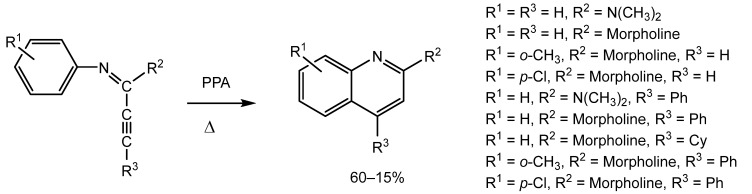
Cyclization of propargylamidines to produce 2-aminoquinolines.

Since the first time these organic compounds were reported, their reactivity has been explored. To begin with, the most-studied reactivity is the treatment of propiolamidines with polyphosphoric acid (PPA) at high temperatures to produce 2-aminoquinolines via a cyclization reaction (Scheme 22) [31].

**Scheme 23:**
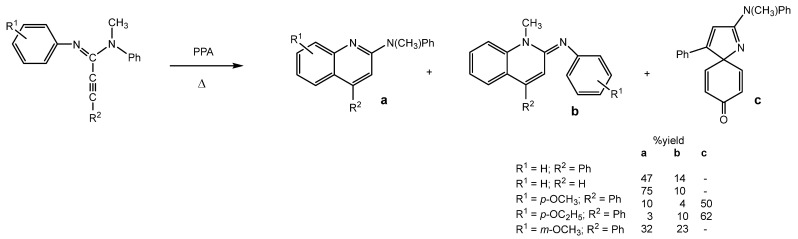
More sophisticated cyclization of propiolamidines with phosphoric acid.

Trying to progress a step further and following this cyclization procedure, it was found that, when the phenylpropiolamidines were substituted with a tertiary nitrogen in the amine group of the CN_2_ core, with one of the substituents being a phenyl ring, a mixture of two isomers was obtained (yields ratio aminoquinoline:iminoquinoline = 47:14), as shown in Scheme 23 [32]. Moreover, when the aromatic ring of the iminic group of the CN_2_ core was substituted in the para-position with either methoxy or ethoxy groups, 2-(N-methylanilino)-4-phenyl-1-azaspiro [4,5]deca-1,3,6,9-tetraen-8-one was formed as the main product (yields ratio aminoquinoline–iminoquinoline–azaspiro = 10:4:50).

**Scheme 24:**
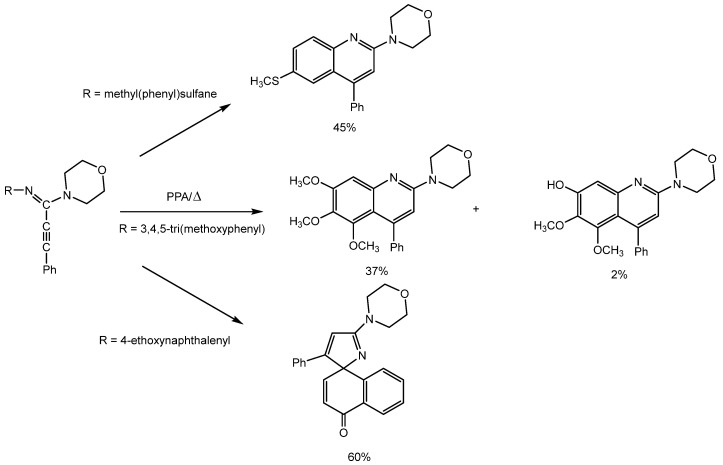
Selective cyclization with polyphosphoric acid.

Lately, this procedure can be customized *à la carte* for phenylpropiolamidines with a morpholine substituent on the aminic nitrogen of the CN_2_ core, depending on the substituent of the iminic nitrogen atom [33]. For instance, when the iminic nitrogen atom was substituted with methyl(phenyl)sulfane, it formed 2-aminoquinoline. A similar product was formed when the substituent was 3,4,5-tri(methoxyphenyl), but, in this case, another minor compound was observed where the methoxy group at position 7 was replaced by a hydroxy function. Conversely, when the amidine was substituted with the 4-ethoxynaphthalenyl group, only the spiro-type compound was produced (Scheme 24).

**Scheme 25:**
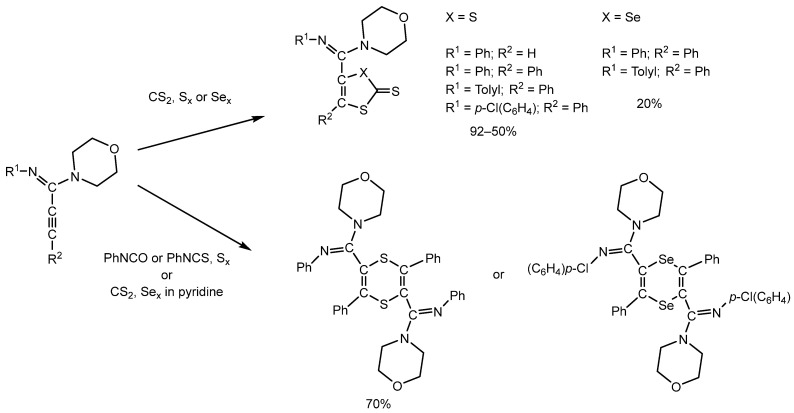
Synthesis of substituted 1,3-dithiole-2-thiones and 1,3-thiaselenole-2-thione.

Keeping the focus on these morpholine amidines, the reactivity of the triple bond of the propargylic unit was studied [62]. The reaction of the propiolamidines with a mixture of carbon disulfide and sulfur in dimethylformamide (DMF) gave 1,3-dithiole-2-thiones. Analogous results were obtained when the reaction was done at 70 °C with selenium or carbon disulfide (Scheme 25). On the other hand, when CS_2_ was replaced by phenylisocyanate or phenylisothiocyanate in the presence of DMF, new 1,4-dithio-compounds were obtained. However, when the reaction was carried out with CS_2_ and selenium in pyridine instead of DMF, the 1,4-diselenine product was produced.

**Scheme 26:**
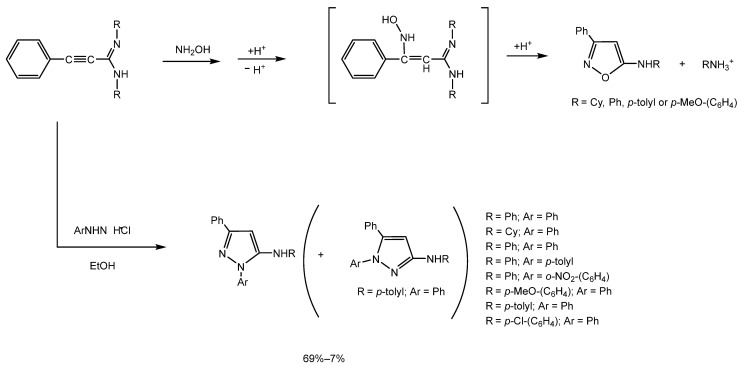
Reactivity with hydroxylamine or hydrazines.

Another interesting finding was made by Fujita et al., who studied the reaction of propiolamidines with hydroxylamines in the presence of acid [35]. This reaction was already known for amidines, where amidoximes were formed and subsequently cyclized to give substituted 3-aminoisoxazoles. However, when the reaction was carried out with propiolamidines, it afforded 5-N-substituted amino-3-phenylisoxazoles. This reaction occurs via the nucleophilic addition of hydroxylamine to the triple bond, followed by cyclization (Scheme 26). On the other hand, when the reaction was carried out with hydrazines, substituted pyrazoles were obtained.

**Scheme 27:**
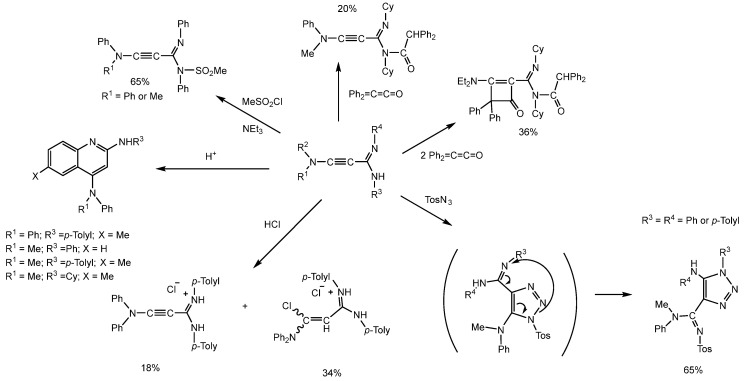
Reactivity of 3-aminopropiooamidines.

Changing the focus to the 3-aminopropiolamidines, an extensive study of their reactivity was conducted by Himbert and Schwickerath (Scheme 27) [36]. The 3-diphenylamino and 3-[methyl(phenyl)amino]propiolamidines could react via the NH motif of the carboxamide group with sulfenes and ketenes. Thus, the reaction with methanesulfonyl chloride (mesyl chloride) formed the corresponding sulfonamides. When the reaction was performed with one equivalent of diphenyl ketene, the subsequent amide was formed. However, employing two equivalents of diphenyl ketene resulted in a double attack: the first equivalent reacted with the NH of the carboxamide, and the second one with the C-C triple bond. Moreover, the addition of hydrochloric acid led to the protonation of the imine N atom of the carboxamide group and also attacked the unsaturation, adding an HCl molecule to the C-C triple bond. When the reaction was carried out with malonic acid, 2,4-diaminoquinolines were obtained, provided that the substituents on the nitrogen atoms of the carboxamide motif were either phenyl or p-tolyl groups. On the other hand, the reaction with tosyl azide gave the corresponding triazole by attacking the triple bond and causing a subsequent rearrangement.

### 3.2. Catalytic Reactivity

Some of the reactivity studies that imply a catalytic reaction are based on a mixture of the primitive compounds (carbodiimides, terminal alkynes, heterocumulenes, etc.) in one pot and one step, usually without any propiolamidine intermediate isolation, although the formation of the amidine is mandatory, as confirmation trials show. However, other reactivity studies begin with a propiolamidine previously synthesized catalytically.

**Scheme 28:**
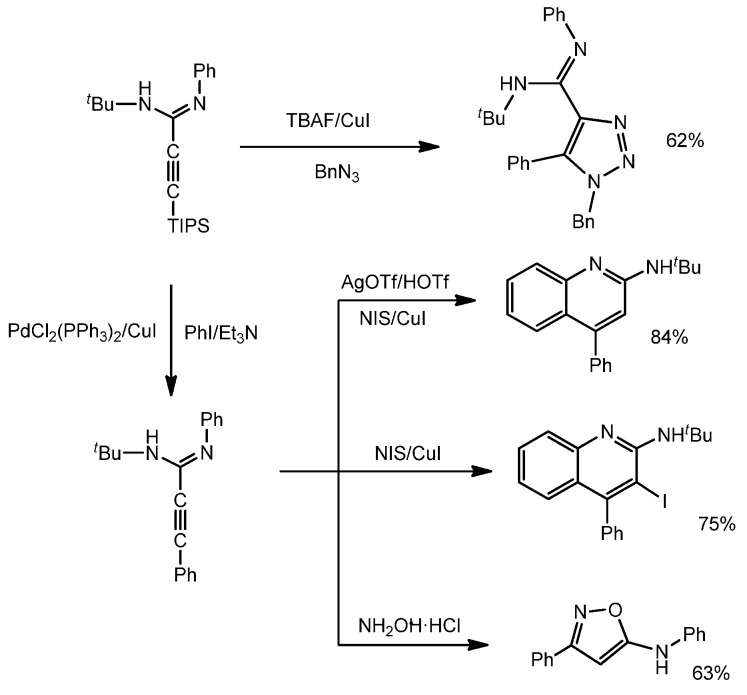
Reactivity of TIPS-propiolamidines.

For those propiolamidines synthesized by a multicomponent reaction from isocyanides, several conversions were performed [50]. Triisopropylsilyl groups could be easily hydrolyzed and replaced by a phenyl group via a PdCl_2_(PPh_3_)_2_/CuI-catalyzed Sonogashira coupling reaction of propiolamidine with iodobenzene. This new propiolamidine was cyclized by AgOTf to afford 2-aminoquinoline. Furthermore, by using N-iodosuccinimide as an electrophile, 3-iodo-2-aminoquinoline can be obtained via electrophilic cyclization catalyzed by CuI. Additionally, the reaction of phenylpropiolamidine with a slight excess of NH_2_OH·HCl produced N,3-diphenylisoxazol-5-amine. TIPS-propiolamidines could also be transformed into triazoles by a click reaction with benzyl azide catalyzed by CuI (Scheme 28).

**Scheme 29:**
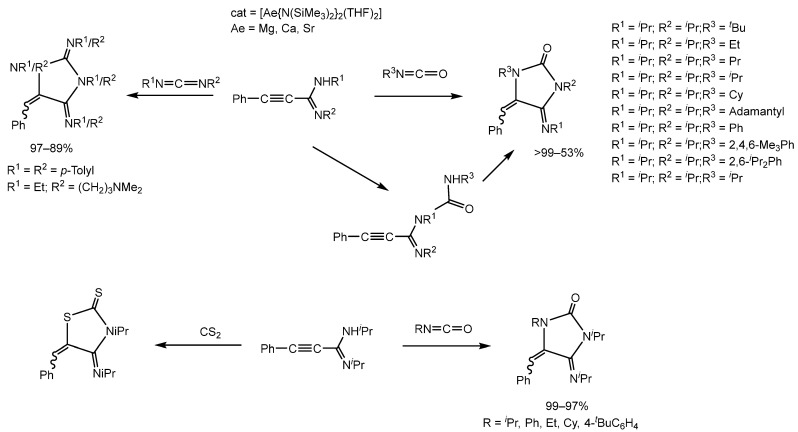
Catalytic synthesis of imidazolidine-2-ones, thiazolidine-2-thione, and imidazolidine-2-thiones.

Another interesting transformation of these compounds was the catalytic formation of imidazolidine-2-ones [63]. In this vein, effective alkaline earth bis(amide) precatalysts were studied in the assembly reaction of propiolamidines and heterocumulenes (Scheme 29). The cyclization reaction with isocyanates occurred via a nucleophilic attack of the NH fragment of the carboxamide on the electrophilic carbon of the isocyanate, producing a formal urea insertion product. Although Mg and Ca precatalysts worked, Sr showed the best conversion. However, when the phenyl group of the alkynyl chain was replaced with a t-Bu group, the cyclization reaction did not take place, resulting in the urea insertion compound as the sole product.

Later, this procedure that employed a Sr catalyst was extended to other aromatic groups in the alkynyl chain, such as o-tolyl, p-tolyl, 1-naphthyl, 3-FC_6_H_4_, 4-FC_6_H_4_, 4-ClC_6_H_4_, 4-MeOC_6_H_4_, 4-CF_3_C_6_H_4_, 3-pyridinyl, and 3-thiophenyl, while keeping iPr groups in the CN_2_ core. Furthermore, other heterocumulenes like CS_2_ or isothiocyanates were used, giving the corresponding thiazolidine-2-thione and imidazolidine-2-thiones, respectively, as shown in Scheme 29 [64].

**Scheme 30:**
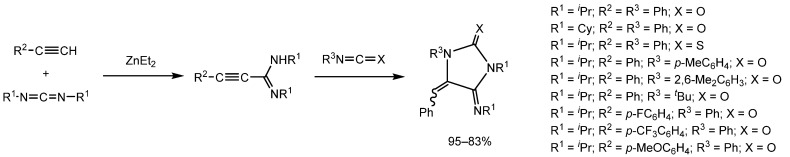
Catalytic synthesis of imidazolidine-2-ones and thiazolidine-2-thione catalyzed by ZnEt_2_.

For this same approach, our research group reported the use of simple and versatile ZnEt_2_ for the cyclization of propiolamidines with isocyanates and thioisocyanates [46]. This reaction consisted of two steps, the first being the hydroalkynylation reaction of the carbodiimide to give the propiolamidine and the second being the insertion of isocyanate/thioisocyanate into the N-H bond, followed by cyclization to form the corresponding imidazolidine-2-ones and thiazolidine-2-thiones without any intermediate purification/isolation step (Scheme 30).

**Scheme 31:**
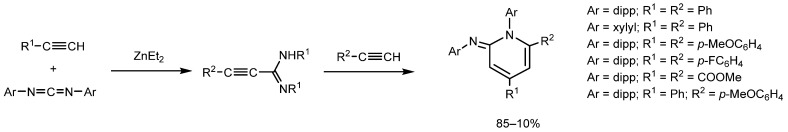
Catalytic synthesis of 2-iminopyridine catalyzed by ZnEt_2_ (dipp = diisopropylphenyl).

Finally, in line with this approach, our research group recently further investigated the cyclization reaction of propiolamidines and terminal alkynes, catalyzed by ZnEt_2_ [65]. The reaction of propiolamidines with equimolar quantities of terminal alkynes resulted in the formation of 2-iminopyridines after heating the reaction at 120 °C for 4 days. Moreover, the procedure was extended to the catalytic formation of propiolamidines and subsequent cyclization in one step, by simply adding one equivalent of carbodiimide and two equivalents of alkyne. However, this reaction achieved good conversion with N,N′-bis(2,6-diisopropylphenyl)carbodiimide, yielded poorly with N,N′-bis(2,6-dimethylphenyl)carbodiimide, and was completely inhibited with any other aromatic or aliphatic carbodiimide. Regarding the alkyne, both substituted aromatic and ester derivatives were well-tolerated (Scheme 31).

**Scheme 32:**
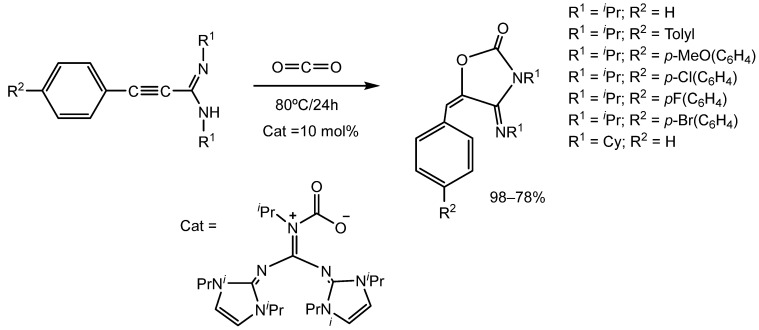
Catalytic synthesis of into 4-iminooxazolidine-2-one derivates by a CO_2_ superbasic guanidine adduct.

A new and green approach was reported by Zhou and colleagues [66]. They demonstrated the viability of capturing CO_2_ with propiolamidines and subsequent catalytic transformation into 4-iminooxazolidine-2-one derivates by a CO_2_ superbasic guanidine adduct. This reaction requires a 10mol% catalyst loading at 80 °C for 24 h in DMSO to give the products in good yields (Scheme 32).

## 4. Conclusions

Propiolamidines can be synthesized using the catalytic hydroalkynylation of carbodiimides with terminal alkynes. In these reactions, catalysts based on the rarely available rare earth elements stand out compared to those based on other metals. The search for simpler systems based on more abundant and even biocompatible metals needs to be pursued further. Developing new catalytic methodologies that improve the functional group tolerance of the catalysts could further enhance the synthesis, allowing for the incorporation of a wider range of substituents on the propiolamidine framework.

Alternatively, less atom-economic stoichiometric methods have been described, based on a wide variety of substrates. It is also necessary to move towards more sustainable alternatives, involving green solvents or even no solvents at all. In either case, detailed studies using techniques like Density Functional Theory can help to gain a deeper understanding of these processes.

Exploring the reactivity of propiolamidines in the formation of more complex molecules, such as heterocycles with biological activity, is an exciting area for future research. This involves not only developing new synthetic routes but also investigating the potential applications of these compounds.

## Data Availability

Data are contained within the article.
